# New Medical Exchanges

**Published:** 1859-02

**Authors:** 


*New Medical Exchanges.*—Since our last issue we have received quite a number of new exchanges, which we hope may be able to live on the troubled sea on which they are embarked. Our friend, the New Orleans Medical and Surgical Journal, has been somewhat enlarged, a gratifying evidence of its temporal welfare. The following are the new Journals which we have received:

*Semi-Monthly Medical News.* Edited by S. M. Bemis, M. D., Prof. of Clinical Medicine, University of Louisville, and J. W. Benson, M. D., Prof. of Descriptive and Surgical Anatomy, University of Louisville.

The first number has a good appearance, in point of mechanical execution, and presents articles of value, from the pens of Drs. Metcalfe, J. B. Flint, Wyble, and J. L. Smith.

*The Louisville Medical Gazette.* Edited by J. L. Frazer, M. D.

This is another journal, published semi-monthly, in the same city. We wish them both success, if possible.

*The Marysville Medical and Surgical Reporter.* Edited and published by Lorenzo Hubbard, M. D., and B. H. Teed, M. D.; containing original Essays on Medical Subjects, and reports of important cases occurring in their own practice. "New Lights often come through Cracks in the Tiling." Vol. 1, No. 1. November, 1858. San Francisco. 8vo., pp. 14.

The above Journal appears to us to be a medium for the self-glorification of the editors, Drs. Hubbard and Teed. It contains only original essays by them, and cases from their *own practice.* However much this may increase their practice in their own region, we do not think it will be the means of earning much glory for them abroad. It consists of fourteen pages, published quarterly, at the moderate price of $3 a year, or $1 per quarter, about seven cents a page; this is about twenty-one times dearer than the ordinary Journals.

*The St. Joseph Journal of Medicine and Surgery*: A bi-monthly Journal, devoted to the advancement of Practical Medicine and Surgery, under the auspices and supervision of the St. Joseph Medical Society. Editorial Committee: J. H. Crane, M. D.; O. B. Knole, M. D., and G. C. Catlett, M. D.

This is the third number of an excellent Journal, published at St. Joseph, Mo. The editor complains that his editorial brethren have not rendered him the courtesy of an exchange. We can only say, for our excuse, that this is the first number which we have seen. We gladly put it on our exchange list.

---

*Dr. Horace Green.*—Our readers may be aware of the excitement which has been made in New York over the death of a Mr. Whitney, the only heir to an immense estate, which was alleged to have been caused by the treatment employed by Dr. Green, *i. e.*, the operation with which he has so closely identified himself, that of tubing the larynx. We are glad to learn that the investigations into the matter by the academy have been completed, and it is conclusively shown that the operation had no instrumentality in producing the fatal event; that there was no rupture of the trachea, as was currently reported, and that the operation was performed but once, and that eight days before death. The testimony before the academy is summed up in the New York Times, which takes a high-toned and honorable view of the alleged conduct of Dr. Mott and Dr. Beales. According to the "Times," and this paper professes to report the proceedings of the academy, Drs. Mott and Beales allowed the rumors, which have been so rife, to circulate on the bare assertion of the patient, who was quite ill, that Dr. Green had killed him by putting a tube in his throat. These gentlemen did not acquaint Dr. Green with this fact; the patient died and they made a post mortem examination, to which Dr. Green was not invited, and even when they discovered no injury to the air passages, they made no movement towards contradicting the rumors.

We have not the space to discuss minutely the merits of this matter, but are convinced from our examination of the facts, that Dr. Green's treatment had nothing to do with the death of Mr. Whitney. If the conduct of Drs. Mott and Beales be as is stated by the "Times," no comment is neccesary. There could be no greater and more flagrant violation of professional honor. Dr. Green has courted an investigation and has been most amply vindicated.

